# Comparative analysis of genetic testing utilization rates among people with and without disabilities in South Korea from 2016 to 2019, focusing on malignant neoplasms: A national population‐based study

**DOI:** 10.1002/cam4.7102

**Published:** 2024-05-06

**Authors:** Gwanwook Bang, Minji Park, Jeong‐yeon Seon, So‐Youn Park

**Affiliations:** ^1^ Department of Medical Education and Humanities, College of Medicine Kyunghee University Seoul South Korea; ^2^ Disability Health Research Center of Kyunghee University Seoul South Korea; ^3^ Kyunghee University Hospital at Gangdong Seoul South Korea; ^4^ Health Insurance Research Institute, National Health Insurance Service Wonju South Korea

**Keywords:** cancer, disability, genetic testing, health policy, South Korea

## Abstract

**Introduction:**

Oncogene testing is widely used to detect or direct cancer treatments. Compared to people without disabilities, people with disabilities in Korea have a lower cancer incidence rate but a fivefold higher cancer mortality rate, implying delayed detection.

**Methods:**

We used an administrative database combining disability status and care utilization to analyze every case of cancer‐related genetic testing paid for by the National Health Insurance Services of Korea between 2016 and 2019. We first compared percentages of individuals who had taken a registered genetic test by their disability statuses. We then compared the most frequently utilized tests between individuals with and without disabilities.

**Results:**

Korean citizens, 175,000 in total, underwent at least one of the 192 registered cancer‐related genetic tests between 2016 and 2019. People with disabilities utilized these genetic tests at higher rates than those without disabilities, regardless of sex or age. Among people aged ≥40 years, lung and colorectal cancer‐related tests were most frequently utilized, regardless of disability status.

**Conclusion:**

Although the cancer‐related genetic test uptake rate is higher among people with disabilities than among those without disabilities, it is still possible that information on these tests is not as readily available to people with disabilities. Therefore, it is imperative for the government to actively devise strategies to enhance national cancer screening rates among people with disabilities.

## 1 INTRODUCTION

Just 30 years ago, cancer resembled a death sentence.^1^ The fatality rate of cancer has considerably decreased in the search for a cure. However, several types of cancer remain fatal and are yet to be overcome by modern medicine. The risk of certain types of cancer is higher in modern times because of factors such as smoking, air and water pollution, and lifestyle changes.^2^

The Korean National Health Insurance statistics confirm this trend. According to Statistics Korea, cancer was the leading cause of death in 2021 among Koreans (161.1/100,000 persons), followed by heart disease (61.5/100,000 persons). These numbers differed by more than 2.5 times.^3^ According to the Korea Central Cancer Registry, the number of new patients with cancer in 2020 was 247,952, and the age‐standardized cancer incidence has been steadily increasing since 2015.^4^ Regarding the incidence rate of each carcinoma, lung cancer ranked first for men and fourth for women, while colorectal cancer ranked fourth for men and third for women, both within the top four for both men and women, although the rankings differ by sex.^5^

People with disabilities have higher risks of cancer, not only in Korea but also worldwide.^6^, ^7^, ^8^ In fact, cancer is the main cause of death in people with disabilities in Korea, who comprise a medically vulnerable group. The cancer mortality and prevalence rates among people with disabilities are 1.05 and 5.02 times higher, respectively, than those among people without disabilities, despite the incidence rate of people with disabilities being marginally lower, at 0.90 times that of people without disabilities.^9^, ^10^ Similarly, people with severe disabilities (Grades 1–3) show higher mortality rates than those with mild disabilities (Grades 4–6), despite the incidence rate being marginally lower.

The National Health Insurance Service (NHIS) of Korea covered genetic testing, one of the advanced medical technologies related to cancer on a national scale in order to reduce the burden of cancer. This genetic testing includes various purposes such as cancer screening, diagnosis, and treatment. Since the genetic testing covered in health insurance is supported either fully or partially by the insurance for people with disabilities, it provides excellent medical support for them to lower the burden related to cancer. However, separate from medical expense support, the discussion on whether disabled individuals are actually utilizing cancer‐related genetic testing through medical cost support services is distinct.

There are various types of genetic testing, including germline genetic testing, which can identify genetic mutations present in an individual is germ cells, and somatic genetic testing, which can identify genetic mutations present in non‐germ cells. In this study, we referred to these types collectively as genetic testing. Genetic testing is utilized for various purposes such as early detection of cancer, identifying risk factors, and guiding treatment.^11^, ^12^, ^13^

Genetic testing detects specific harmful mutations in genes that increase the risk of developing cancer or informs genetic susceptibility to specific types of cancer that are hereditary. In addition to predicting the risk of cancer, tumor DNA sequencing can contribute to increasing the cancer survival rate by allowing customized treatment of the cancer cells according to gene changes of the said cancer cells. For example, of the three types of RAT Sarcoma virus (RAS), the oncogene Kirsten Rat Sarcoma Viral Oncogene Homolog (*KRAS*) is known to have a 40% mutation rate, whereas the Neuroblastoma Rat Sarcoma Viral Oncogene Homolog (*NRAS*) has a mutation rate of approximately 5%–8%.^14^ Medical teams can predict responsiveness to existing targeted therapies by verifying the presence or absence of these mutations. New therapies targeting these mutations are currently under development.

Research on whether individuals with disabilities are effectively utilizing high‐utilization genetic testing like this has not been well conducted thus far. While there are several studies on cancer‐related genetic‐test utilization, disability status is rarely recognized as a factor, either as a motivator or a barrier. To the best of our knowledge, whether genetic‐test utilization patterns differ by disability status is yet to be studied.

Therefore, in this study, we aimed to analyze the entire population using national data sources to identify which genetic tests are actually widely utilized by individuals with disabilities in Korea, as well as to assess the screening rates for cancer among this group. Based on our findings, we intend to propose social and institutional measures to improve earlier detection and better treatment for cancer for people with disabilities.

## 2 MATERIALS AND METHODS

### 2.1 Data sources and sample criteria

As the majority of Korean citizens are enrolled in medical insurance, we used a national administrative dataset, the National Health Insurance Service of Korea (NHIS) and Korean Statistical Information Service (KOSIS), which comprises the entire record of genetic tests utilized by Korean citizens. We also identified individuals with disabilities by their Ministry of Health and Welfare disability registration statuses, leaving little room for measurement error.^15^ Patients who underwent genetic testing between 2016 and 2019 were included in this study. The NHIS database stores the premier data of Korea, and includes information on health insurance qualifications, insurance premiums, diagnoses, and procedure codes of care paid by the national health insurance. We included individuals with Korean citizenship who underwent one of the 192 genetic tests (Appendix) listed in the National Health Insurance Payment Scheme after approval by the National Evidence‐based Healthcare Collaborating Agency of Korea through their New Medical Technology Evaluation. The total number of patients who underwent genetic testing between 2016 and 2019 was 175,330. We studied the annual trends, demographic characteristics, and medical care utilization of these individuals based on their disability status.

Types of genetic tests mainly include those used for various purposes, such as preventing specific rare diseases or diagnosing and treating cancer. The following genetic tests are the main genetic tests in the top five shown in the results of this study.

1. EGFR Gene Test: Genetic test to confirm drug sensitivity in patients with non‐small cell lung cancer (Non‐Small Cell Lung Cancer)
2. KRAS Gene Test: Genetic test to determine treatment policy for patients with colon cancer (colorectal cancer)
3. NRAS Gene Test: Genetic test to check for NRAS gene mutation in patients with colon cancer (colorectal cancer)
4. BRAF Gene Test: Genetic test to predict prognosis of patients undergoing surgery for papillary thyroid cancer (papillary thyroid carcinoma)
5. IGH Gene Test: Genetic test to differentiate between malignant lymphoma and benign lymphoproliferative disease (lymphoproliferative disease)

### 2.2 Statistical analyses

We compared the characteristics of patients who underwent genetic testing and identified the most frequently used method based on their disability status. Sociodemographic characteristics included sex and age (grouped in tens), health insurance premiums, and disability status. Health insurance premium levels reflected the economic well‐being of households. Recipients of medical benefits did not pay insurance premiums and were considered low‐income households. Those who paid health insurance premiums were categorized into five groups: low‐ (first quintile) to high‐income (fifth quintile) households. Disability status depends on registration in the Korea National Disability Registration System.

### 2.3 Ethical considerations

This study complied with the Guidelines on De‐Identification of Personal Data of Korea and was approved by the Institutional Review Board of Kyung Hee University [IRB No. KHSIRB‐20‐074(EA)] as a review exemption. Given that the study used de‐identified data, the board waived the requirement for obtaining informed consent from the participants.

## 3 RESULTS

### 3.1 Descriptive statistics

The number of genetic tests registered in the National Health Insurance payment scheme gradually increased from 92 in 2016 to 181 in 2017 and 192 in 2018. Accordingly, the frequency of genetic testing also increased from 38,239 in 2016 to 56,883 in 2017 and 70,396 in 2018. The number of patients who underwent genetic testing increased from 28,924 in 2016 to 56,118 in 2019. Reflecting these upward trends, the cost of genetic testing claims paid by national health insurance increased from approximately 6.2 billion in 2016 to 9.8 billion in 2017 and 11.8 billion in 2018 (Table 1).

**TABLE 1:** The number of Korean patients who underwent genetic cancer tests according to their sociodemographic characteristics between 2016 and 2019 (Unit: persons).

	2016		2017		2018		2019	
	Number	Proportion (%)	Number	Proportion (%)	Number	Proportion (%)	Number	Proportion (%)
Total	28,924	100	41,739	100	48,549	100	56,118	100
Sex								
Men	16,436	56.8	23,808	57.0	27,090	55.8	30,543	54.4
Women	12,488	43.2	17,931	43.0	21,459	44.2	25,575	45.6
Ages in years								
0–19	702	2.4	1288	3.1	1273	2.6	1322	2.4
20–29	575	2.0	1001	2.4	1186	2.4	1364	2.4
30–39	1141	3.9	1975	4.7	2486	5.1	3037	5.4
40–49	2708	9.4	4085	9.8	4581	9.4	5188	9.2
50–59	5926	20.5	8415	20.2	9369	19.3	10,477	18.7
60–69	8000	27.7	10,824	25.9	12,513	25.8	14,837	26.4
70–79	7273	25.1	10,195	24.4	12,013	24.7	13,676	24.4
80 or older	2599	9.0	3956	9.5	5128	10.6	6217	11.1
Health Insurance Premium								
Medical benefit recipients	1942	6.7	2926	7.0	3459	7.1	4271	7.6
First quintile	3869	13.4	5932	14.2	7217	14.9	9046	16.1
Second quintile	3807	13.2	5256	12.6	6105	12.6	6453	11.5
Third quintile	4470	15.5	6518	15.6	7589	15.6	8747	15.6
Fourth quintile	5894	20.4	8537	20.5	9862	20.3	11,274	20.1
Fiftth quintile	8942	30.9	12,570	30.1	14,317	29.5	16,327	29.1
Disability status								
Not registered	25,340	87.6	36,526	87.5	42,348	87.2	49,060	87.4
Registered	3584	12.4	5213	12.5	6201	12.8	7058	12.6

### 3.2 Status of genetic testing use according to disability type

The analysis included 15 legally defined types of disabilities in Korea. Every year, an increasing number of people with disabilities undergo genetic testing. In 2016, when genetic testing was registered as a reimbursement, it was 0.16%, but in 2019, it approximately doubled to 0.27%. In particular, the usage rate of people with internal organ disabilities was the highest at 0.48%, and the usage rate of people with mental disabilities was the lowest at 0.09%. We could not analyze detailed test types by disability type to prevent personal identification because of the small number of users (Table 2).

**TABLE 2:** Status of genetic testing according to disability type (Unit: persons).

Type***	2016			2017			2018			2019		
	Number of registered people with disabilities*	Number of patients	Ratio (%)**	Number of registered people with disabilities*	Number of patients	Ratio (%)**	Number of registered people with disabilities*	Number of patients	Ratio (%)**	Number of registered people with disabilities*	Number of patients	Ratio (%)**
**Total**	**2,511,051**	**3584**	**0.14**	**2,545,637**	**5213**	**0.20**	**2,585,876**	**6201**	**0.24**	**2,618,918**	**7058**	**0.27**
**Disability of principal external bodily functions**	**2,064,356**	**3120**	**0.15**	**2,612,276**	**4413**	**0.17**	**2,110,587**	**5302**	**0.25**	**2,129,630**	**6049**	**0.28**
Disability of the extremities	1,267,174	1861	0.15	1,254,130	2563	0.20	1,238,532	2952	0.24	1,223,135	3239	0.26
Disability due to brain injury	250,456	389	0.16	252,819	687	0.27	253,083	717	0.28	252,188	812	0.32
Visual disability	252,794	373	0.15	252,632	**482**	0.19	252,957	597	0.24	253,055	703	0.28
Hearing disability	271,843	**462**	0.17	302,003	641	0.21	342,582	977	0.29	377,094	1216	0.32
Speech and language disability	19,409	32	0.16	20,321	40	0.20	20,744	54	0.26	21,485	73	0.34
Facial deformity disability	2680	3	0.11	2692	0	0.00	2689	5	0.19	2673	6	0.22
**Disability of internal organs**	**128,490**	**349**	**0.27**	**134,264**	**569**	**0.42**	**139,529**	**630**	**0.45**	**144,694**	**699**	**0.48**
Disability due to renal failure	78,750	120	0.15	83,562	214	0.26	87,892	242	0.28	92,408	309	0.33
Disability due to heart problems	5507	12	0.22	5399	23	0.43	5304	27	0.51	5266	26	0.49
Disability due to respiratory problems	11,831	39	0.33	11,807	**41**	0.35	11,761	63	0.54	11,522	53	0.46
Disability due to liver disease	11,042	25	0.23	11,843	34	0.29	12,524	42	0.34	13,154	43	0.33
Disability due to ostomy	14,404	142	0.99	14,718	250	1.70	15,027	241	1.60	15,290	253	1.65
Disability due to epilepsy	6956	11	0.16	6935	7	0.10	7021	**15**	0.21	7054	15	0.21
**Mental disability**	**318,205**	**115**	**0.04**	**326,776**	**231**	**0.07**	**335,760**	**269**	**0.08**	**344,594**	**310**	**0.09**
Intellectual disability	195,283	67	0.03	200,903	127	0.06	206,917	162	0.08	212,936	193	0.09
Disability due to autism	22,853	3	0.01	24,698	3	0.01	26,703	5	0.02	28,678	9	0.03
Disability due to mental disorders	100,069	45	0.04	101,175	101	0.10	102,140	102	0.10	102,980	**108**	**0.10**

* The number of registered people with disabilities is based on the Ministry of Health and Welfare's “Status of people with disabilities” data.

** The ratio is the number of genetic test patients compared to the number of registered disabled people (= number of patients/number of registered people with disabilities × 100).

*** The types of disabilities are based on the criteria classified by Korean law.

Boldface is intended to distinguish between each type of disability.

### 3.3 Differences in the percentage of patients using genetic testing according to disability status

A higher proportion of people with disabilities utilized genetic testing than those without disabilities (*p* < 0.001), regardless of sex and age (Table 3). This difference grows every year.

**TABLE 3:** Comparison of the proportions of patients who utilize oncogene genetic tests for persons with and without disabilities (unit: %).

	2016				2017				2018				2019			
	The proportion of patients who utilized oncogene genetic tests (%)			*p*‐value	The proportion of patients who utilized oncogene genetic tests (%)			*p*‐value	The proportion of patients who utilized oncogene genetic tests (%)			*p*‐value	The proportion of patients who utilized oncogene genetic tests (%)			*p*‐value
	All	People without disabilities	People with disabilities		All	People without disabilities	People with disabilities		All	People without disabilities	People with disabilities		All	People without disabilities	People with disabilities	
**All**	**0.06**	**0.05**	**0.14**	<0.001	**0.08**	**0.07**	**0.20**	<0.001	**0.09**	**0.09**	**0.24**	<0.001	**0.11**	**0.10**	**0.27**	<0.001
Sex																
Men	**0.06**	**0.06**	**0.17**	<0.001	**0.09**	**0.08**	**0.23**	<0.001	**0.10**	**0.09**	**0.27**	<0.001	**0.12**	**0.11**	**0.30**	<0.001
Women	**0.05**	**0.05**	**0.11**	<0.001	**0.07**	**0.07**	**0.16**	<0.001	**0.08**	**0.08**	**0.20**	<0.001	**0.10**	**0.09**	**0.22**	<0.001
Ages (years)																
0–19	**0.01**	**0.01**	**0.07**	<0.001	**0.01**	**0.01**	**0.12**	<0.001	**0.01**	**0.01**	**0.10**	<0.001	**0.01**	**0.01**	**0.11**	<0.001
20–29	**0.01**	**0.01**	**0.04**	<0.001	**0.01**	**0.01**	**0.05**	<0.001	**0.02**	**0.02**	**0.05**	<0.001	**0.02**	**0.02**	**0.05**	<0.001
30–39	**0.01**	**0.01**	**0.04**	<0.001	**0.03**	**0.03**	**0.06**	<0.001	**0.03**	**0.03**	**0.09**	<0.001	**0.04**	**0.04**	**0.10**	<0.001
40–49	**0.03**	**0.03**	**0.06**	<0.001	**0.05**	**0.05**	**0.10**	<0.001	**0.05**	**0.05**	**0.12**	<0.001	**0.06**	**0.06**	**0.11**	<0.001
50–59	**0.07**	**0.07**	**0.10**	<0.001	**0.10**	**0.10**	**0.15**	<0.001	**0.11**	**0.11**	**0.16**	<0.001	**0.12**	**0.12**	**0.19**	<0.001
60–69	**0.15**	**0.15**	**0.18**	<0.001	**0.19**	**0.19**	**0.25**	<0.001	**0.21**	**0.21**	**0.28**	<0.001	**0.24**	**0.23**	**0.30**	<0.001
70–79	**0.23**	**0.23**	**0.24**	<0.001	**0.31**	**0.31**	**0.32**	<0.001	**0.35**	**0.35**	**0.37**	<0.001	**0.39**	**0.38**	**0.43**	<0.001
80 or older	**0.18**	**0.18**	**0.18**	0.140	**0.25**	**0.26**	**0.24**	<0.001	**0.31**	**0.31**	**0.31**	0.311	**0.34**	**0.35**	**0.33**	<0.001

*Note*: Numbers calculated by the authors based on the Population and Housing Census by Statistics Korea and Health Statistics of the People with Disabilities by the Ministry of Health and Welfare of Korea; *p*‐values are for *t*‐tests for differences between two proportions among people with and without disabilities.

Boldface is intended to distinguish between each type of disability.

### 3.4 Frequently utilized genetic testing according to disability status

We analyzed the five most commonly utilized genetic tests across different age groups. Notably, leukemia was the predominant diagnosis among individuals with disabilities aged 39 years or younger. In people with and without disabilities, EGFR gene tests related to lung cancer and KRAS and NRAS gene tests related to colorectal cancer appeared as the top genetic test items among people aged ≥40 years (Table 4).

**TABLE 4:** The most frequently used genetic tests are based on disability status and age.

Ages (years)	Disability status	Ranked				
		First (relevant disease)	Second (relevant disease)	Third (relevant disease)	Fourth (relevant disease)	Fifth (relevant disease)
0–19	Without disabilities	WT1 test (acute myelocytic leukemia)	IGH test (lymphoproliferative disease)	TRG test (lymphoproliferative disease)	IGK test (lymphoproliferative disease)	RNF213 test (moyamoya disease)
	With disabilities	IGH test (lymphoproliferative disease)	WT1 Mutation testing (acute myelocytic leukemia)	IGK Mutation testing (lymphoproliferative disease)	TRG Mutation testing (lymphoproliferative disease)	CHD7 Mutation testing (charge syndrome)
20–29	Without disabilities	WT1 Mutation testing (acute myelocytic leukemia)	AALC Mutation testing (acute myelocytic leukemia)	IGH Mutation testing (lymphoproliferative disease)	BRAF Mutation testing (papillary thyroid carcinoma)	RNF213 Mutation testing (moyamoya disease)
	With disabilities	BAALC Mutation testing (acute myelocytic leukemia)	WT1 Mutation testing (acute myelocytic leukemia)	JAK2 Mutation testing (chronic myeloproliferative disease)	TBP Mutation testing (Spinocerebellar Ataxia Type 17)	ATXN8 Mutation testing (Spinocerebellar Ataxia Type 8)
30–39	Without disabilities	JAK2 Mutation testing (chronic myeloproliferative disease)	WT1 Mutation testing (acute myelocytic leukemia)	MTHFR Mutation testing (hyperhomocysteinemia)	BAALC Mutation testing (acute myelocytic leukemia)	EGFR Mutation testing (Non‐Small Cell Lung Cancer)
	With disabilities	TBP Mutation testing (Spinocerebellar Ataxia Type 17)	RNF213 Mutation testing (Moyamoya disease)	BAALC Mutation testing (Acute myeloblastic leukemia) JAK2 Mutation testing (Chronic myeloproliferative disease)	‐	ATXN8 Mutation testing (Spinocerebellar Ataxia Type 8)
40–49	Without disabilities	EGFR Mutation testing (Non‐Small Cell Lung Cancer)	BRAF Mutation testing (papillary thyroid carcinoma)	KRAS Mutation testing (colorectal cancer)	WT1 Mutation testing (Acute myelocytic leukemia)	IGH Mutation testing (lymphoproliferative disease)
	With disabilities	KRAS Mutation testing (colorectal cancer)	EGFR Mutation testing (Non‐Small Cell Lung Cancer)	WT1 Mutation testing (Acute myelocytic leukemia)	JAK2 Mutation testing (Chronic myeloproliferative disease)	BAALC Mutation testing (Acute myelocytic leukemia)
50–59	Without disabilities	EGFR Mutation testing (Non‐Small Cell Lung Cancer)	KRAS Mutation testing (colorectal cancer)	BRAF Mutation testing (papillary thyroid carcinoma)	NRAS Mutation testing (colorectal cancer)	IGH Mutation testing (lymphoproliferative disease)
	With disabilities	EGFR Mutation testing (Non‐Small Cell Lung Cancer)	KRAS Mutation testing (colorectal cancer)	NRAS Mutation testing (colorectal cancer)	BRAF Mutation testing (papillary thyroid carcinoma)	TBP Mutation testing (Spinocerebellar Ataxia Type 17)
60–69	Without disabilities	EGFR Mutation testing (Non‐Small Cell Lung Cancer)	KRAS Mutation testing (colorectal cancer)	NRAS Mutation testing (colorectal cancer)	BRAF Mutation testing (papillary thyroid carcinoma)	IGH Mutation testing (lymphoproliferative disease)
	With disabilities	EGFR Mutation testing (Non‐Small Cell Lung Cancer)	KRAS Mutation testing (colorectal cancer)	NRAS Mutation testing (colorectal cancer)	BRAF Mutation testing (papillary thyroid carcinoma)	IGH Mutation testing (lymphoproliferative disease)
70–79	Without disabilities	EGFR Mutation testing (Non‐Small Cell Lung Cancer)	KRAS Mutation testing (colorectal cancer)	NRAS Mutation testing (colorectal cancer)	BRAF Mutation testing (papillary thyroid carcinoma)	IGH Mutation testing (lymphoproliferative disease)
	With disabilities	EGFR Mutation testing (Non‐Small Cell Lung Cancer)	KRAS Mutation testing (colorectal cancer)	NRAS Mutation testing (colorectal cancer)	BRAF Mutation testing (papillary thyroid carcinoma)	IGH Mutation testing (lymphoproliferative disease)
80 or older	Without disabilities	EGFR Mutation testing (Non‐Small Cell Lung Cancer)	KRAS Mutation testing (colorectal cancer)	NRAS Mutation testing (colorectal cancer)	BRAF Mutation testing (papillary thyroid carcinoma)	IGH Mutation testing (lymphoproliferative disease)
	With disabilities	EGFR Mutation testing (Non‐Small Cell Lung Cancer)	KRAS Mutation testing (colorectal cancer)	NRAS Mutation testing (colorectal cancer)	BRAF Mutation testing (papillary thyroid carcinoma)	CYP2C9 Mutation testing (CYP2C9 metabolizing drugs*)

* Include warfarin, phenytoin, fluoxetine, sertraline, and losartan.

### 3.5 Cancer screening rates for people with disabilities by cancer type

**FIGURE 1:**
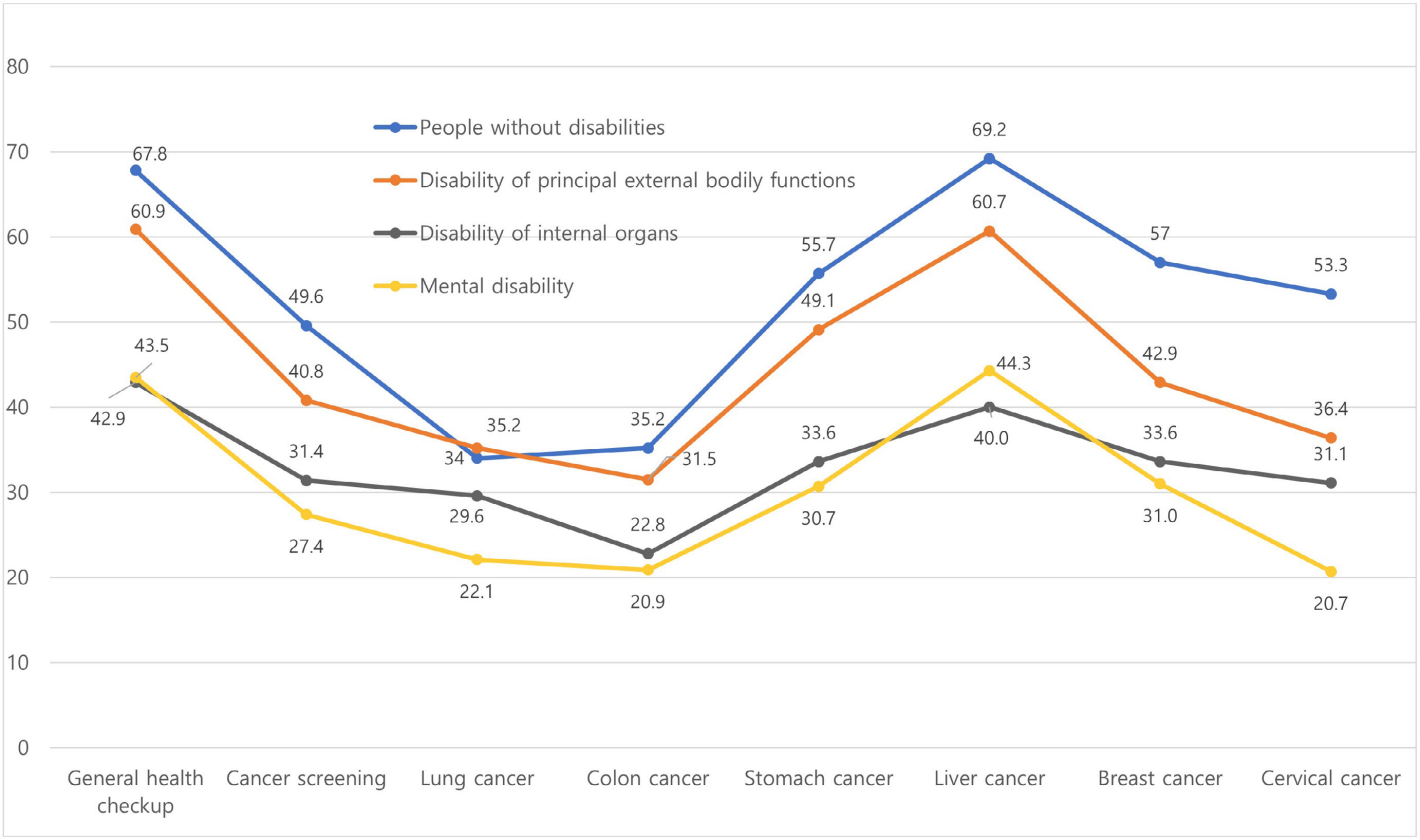
Cancer screening rates for people with disabilities by cancer type (%).

In individuals with disabilities, the rate of cancer screening was generally low for all types except lung cancer. When considering specific types, excluding cervical cancer, which was only screened in women, lung and colon cancers had the lowest screening rates. There was a notable trend: younger individuals, those with lower income, more severe disabilities, or a shorter duration since disability registration tended to have lower cancer screening rates. Particularly, people with mental disabilities recorded the lowest screening rates. Individuals with internal organ disabilities followed the low screening rates, and then those with disabilities affecting major external body functions (Table 5) (Figure 1).

**TABLE 5:** Cancer screening rates for people with disabilities by cancer type.

		General health checkup	Cancer screening	Lung cancer	Colon cancer	Stomach cancer	Liver cancer	Breast cancer	Cervical cancer
		Examination rate (%)*							
Total	Total	57.9	39.2	34.4	30.3	46.7	55.9	41.4	34.2
Control group	People without disabilities	67.8	49.6	34.0	35.2	55.7	69.2	57.0	53.3
Sex	Men	60.4	40.9	34.7	32.5	48.7	56.4	‐	‐
	Women	54.2	37.1	24.8	27.7	44.1	54.9	41.4	34.2
Age in years	0–19	85.7	‐	‐	‐	‐	‐	‐	‐
	20–29	47.5	15.3	‐	‐	‐	‐	‐	15.3
	30–39	54.4	31.1	‐	‐	‐	‐	‐	31.1
	40–49	60.3	48.3	‐	‐	44.0	57.4	43.5	39.7
	50–59	60.9	38.2	31.3	28.1	47.0	55.7	49.7	46.6
	60–69	66.1	47.2	36.6	37.7	56.0	61.4	57.0	51.3
	70–79	62.6	42.9	32.8	34.6	53.1	55.5	46.5	37.2
	80 or older	35.5	21.9	‐	16.3	25.7	34.2	17.3	10.7
Categories of Policyholders	Employer‐provided policyholders	65.3	44.6	38.5	34.8	53.6	62.7	46.7	39.1
	Locally provided policyholders	51.1	39.0	33.9	30.3	45.9	59.0	41.4	34.6
	Persons eligible for medical care	33.5	24.0	21.9	17.4	28.2	39.2	26.9	21.3
Quartile by insurance premium	1st quartile	63.7	45.4	38.8	37.0	52.7	64.6	48.7	40.6
	2nd quartile	65.4	47.3	41.3	38.3	55.1	66.3	50.1	43.0
	3rd quartile	63.4	44.5	37.4	34.5	53.1	63.6	47.0	40.2
	4th quartile	56.3	38.3	31.1	28.6	47.2	55.6	39.8	32.3
Urbanicity	Large city	57.7	38.4	34.2	29.6	46.4	56.1	42.3	37.0
	Small and medium‐sized city	58.5	39.9	36.9	31.2	46.8	57.1	41.0	33.7
	Rural	56.2	38.7	25.7	28.9	47.2	50.9	40.3	28.8
Region	Seoul	55.2	36.6	27.3	29.3	44.0	49.9	41.5	37.1
	Busan	57.3	37.8	33.8	28.4	46.1	60.0	41.2	34.8
	Daegu	57.0	35.9	33.0	23.6	45.4	58.9	37.3	33.2
	Incheon	61.1	42.6	45.2	35.5	49.5	58.6	45.0	38.2
	Gwangju	59.6	41.6	39.6	30.7	49.9	58.2	45.1	38.4
	Daejeon	61.8	41.3	35.9	30.8	50.7	60.2	47.0	39.6
	Ulsan	63.5	41.1	36.6	31.2	48.9	60.6	42.7	39.9
	Sejong	63.9	42.9	33.9	29.1	52.0	62.7	41.8	36.6
	Kyeonggi	58.6	39.8	33.8	32.3	46.2	54.7	41.9	35.9
	Gangwon	58.4	40.1	33.6	32.4	47.4	52.6	41.1	33.0
	Chungcheongbuk	60.0	40.4	29.8	30.3	49.8	53.4	43.7	34.3
	Chungcheongnam	57.2	39.3	31.2	30.8	46.6	52.9	40.5	31.2
	Jeollabuk	58.8	40.5	30.8	30.8	48.4	54.1	41.7	32.9
	Jeollanam	56.9	40.8	41.4	30.2	48.1	57.3	42.3	29.7
	Gyeongsangbuk	56.2	38.7	37.1	29.4	45.6	56.9	36.9	28.3
	Gyeongsangnam	58.0	39.3	36.0	29.1	47.5	60.4	39.8	30.0
	Jeju	52.7	33.3	45.6	23.9	40.2	56.2	38.2	30.6
Disability type	**Disability of principal external bodily functions**	60.9	40.8	35.2	31.5	49.1	60.7	42.9	36.4
	Disability of the extremities	65.6	44.9	35.3	34.6	54.1	63.5	48.7	41.4
	Disability due to brain injury	38.2	25.0	28.9	19.7	28.2	44.5	22.0	19.3
	Visual disability	62.3	41.5	35.3	31.6	50.1	61.7	45.1	39.4
	Hearing disability	57.3	37.5	37.4	29.4	45.2	57.4	36.0	29.5
	Speech and language disability	50.6	32.0	25.0	24.6	35.9	51.1	36.6	33.3
	Facial deformity disability	64.4	43.5	31.8	32.6	52.2	62.5	48.9	44.5
	**Disability of internal organs**	42.9	31.4	29.6	22.8	33.6	40.0	33.6	31.1
	Disability due to renal failure	39.2	29.3	29.3	20.4	30.6	45.1	30.8	29.6
	Disability due to heart problems	53.5	38.3	27.6	31.4	41.5	41.1	43.9	38.7
	Disability due to respiratory problems	46.7	33.2	26.5	26.6	36.3	52.5	35.9	33.9
	Disability due to liver disease	55.4	40.5	29.2	31.1	44.4	33.4	47.4	44.7
	Disability due to ostomy	46.0	29.6	35.8	22.0	34.8	46.8	31.8	22.6
	Disability due to epilepsy	49.3	38.4	27.8	27.8	44.3	51.8	47.7	41.3
	**Mental disability**	43.5	27.4	22.1	20.9	30.7	44.3	31.0	20.7
	Intellectual disability	46.7	26.6	17.9	20.4	31.2	45.5	29.6	16.8
	Disability due to autism	40.7	8.3	0.0	16.0	31.3	50.0	23.5	2.8
	Disability due to mental disorders	38.1	28.5	23.6	21.2	30.3	43.1	32.2	26.6
Disability severity	Severe (level 1 ~ 3)	46.1	30.4	29.6	23.3	35.1	49.0	30.9	24.8
	Mild (level 4 ~ 6)	64.1	43.4	36.0	33.5	52.3	59.4	46.2	39.2
Years since disability registration	Less than 10 years	52.6	36.0	34.5	27.8	42.4	50.3	37.6	31.6
	Between 10 and 20 years	60.1	40.7	34.6	31.3	48.8	58.0	43.4	35.8
	More than 20 years	59.0	39.4	34.1	30.9	46.8	58.0	40.8	33.2

Note The data were prepared as of 2020 because the country has not yet provided the data for 2021.

* The examination rate was calculated based on the number of people subject to examination/number of people examined.

## 4 DISCUSSION

This study aims to achieve two objectives. First, it seeks to determine the frequency and types of genetic tests utilized by individuals with disabilities, specifically focusing on identifying the types of cancer that are most needed among this population. Second, it aims to evaluate the cancer screening rates among individuals with disabilities for the specific types of cancer identified earlier.

The results of the first objective are as follows. The inclusion of genetic tests in the National Health Insurance Service is increasing, leading to a rise in the frequency of genetic testing. The study findings revealed that the national health insurance expenditure on genetic testing amounted to 6.2 billion in 2016, 9.8 billion in 2017, and approximately 11.8 billion in 2018.

In Korea, the government continues to supplement the list of genetic tests on national health insurance benefits to meet the public demand for healthcare and promote general health. Although numerous citizens benefit from these tests, people with disabilities might not benefit as much because of difficulties in accessing and understanding emerging medical technologies.^16^

People with disabilities aged <40 years primarily undergo tests for genetic diseases, such as lymphoproliferative diseases, acute myelocytic leukemia, and chronic myeloproliferative diseases. Among those aged ≥40 years, who accounted for 88.4% of the total number of people with disabilities, the top three most frequently used tests were colorectal and lung cancer‐related (KRAS, NRAS, and EGFR).

Cancer is the most common cause of death for people with disabilities in Korea. The crude death rate for the entire population because of cancer is 161.1/100,000 people, whereas the number for people with disabilities is 3.8 times higher at 604.3/100,000 people. In other words, cancer is more lethal in individuals with disabilities than in those without.^9^ This result is consistent with findings in studies conducted in other countries.^6^, ^17^ Our findings show that among people with disabilities aged 40 years and older, genetic tests related to colorectal and lung cancer are the most frequently used. These numbers imply a critical need to mitigate cancer risk in individuals with disabilities at various stages, including prevention, diagnosis, treatment, and care.

Based on these findings, we analyzed the cancer screening rates among individuals with disabilities focusing on two specific types of cancer selected from among numerous cancers: lung cancer and colorectal cancer.

As of 2020, the cancer screening rate among people with disabilities was 39.2%, which was lower than the cancer screening rate of 55.3% among people without disabilities in South Korea.^18^ Among different types of disabilities, people with brain lesions, intellectual disabilities, and those on the autism spectrum have lower cancer screening rates.^19^ Regarding cancer type, the colorectal cancer screening rate through methods such as colonoscopy or fecal occult blood testing was 30.3% for people with disabilities and 35.2% for people without disabilities, both of which are lower than all averages for cancer screening rates.^20^ The percentages of lung cancer were 34.0% and 34.4% for people with and without disabilities, respectively, and a similar trend can also be found in studies conducted in other countries.^21^

The Korean government introduced the National Cancer Screening Program in 2002 to select six types of cancer to reduce cancer mortality through early cancer detection because of this risk.^22^ Among them, colon cancer and lung cancer are also included. However, the risk of cancer remains high among people with disabilities. One of the reasons for this is the lower uptake rate for cancer screening by people with disabilities due to multiple barriers.^23^, ^24^

For example, in colonoscopy, which is mainly used in colon cancer screening, people with disabilities face barriers such as limitations in changing positions, difficulties in communication, lack of mobility equipment, etc. Significant variances exist in the obstacles faced during cancer screening, which are influenced by the type and severity of disability.^25^, ^26^, ^27^, ^28^ However, governmental institutional support in addressing these challenges remains notably insufficient.

It is crucial to improve early detection methods that are specifically designed for this population to reduce cancer‐related risks among individuals with disabilities. Particularly, those with reduced cognitive abilities, such as individuals with brain lesions or those on the autism spectrum, could significantly benefit from specially tailored promotional efforts. These efforts might involve providing information in language formats that align with their cognitive understanding levels. For example, we can proactively identify and engage with high‐risk groups within the disabled population, especially those with a familial history of cancer. We can foster greater awareness and participation by offering them comprehensive information about the advantages of early screening and genetic testing.

Additionally, the development and implementation of health literacy programs are vital. These programs should aim to demystify complex medical concepts, presenting them in a straightforward and accessible manner. Emphasizing the workings, benefits, and role of genetic testing in early cancer detection in an easily comprehensible language can further promote screening uptake.

## 5 LIMITATIONS

In Korea, individuals with disabilities are often overlooked in medical research and healthcare policy debates.^29^ Specifically, research focusing on genetic testing for people with disabilities has been notably absent, only garnering attention recently. While genetic testing holds significant relevance for this demographic, it presents various challenges that must be addressed.

Although this study offers valuable insights, it has certain limitations. The primary constraint is that the findings, although obtained from the National Health Insurance Service (NHIS) data in the Republic of Korea, are based on a relatively small sample of individuals with disabilities. Consequently, these results may not be widely generalizable. Second, the low number of participants who underwent genetic testing, relative to the diversity of tests available, limited the depth of our analysis. Detailed analysis was further constrained to avoid the risk of individual identification. Despite these limitations, the study is noteworthy as it pioneers the exploration of genetic testing needs among people with disabilities in Korea. Additionally, the results are discussed in the context of Korea's current health and welfare policies. Further research, incorporating data from other countries, is essential to enhance understanding and substantiate our findings.

## 6 CONCLUSION

This study represents a pioneering analysis of the utilization of genetic testing among individuals with disabilities in Korea, identifying the specific diseases for which such testing is most commonly employed in this group. As far as we are aware, this marks the first study in South Korea to explore the rate of genetic testing for cancer using national data among people with disabilities.

Our research made significant findings using data from the National Health Insurance database and supplemented by other reliable sources like Statistical Korea and the National Office of Statistics. Notably, the most crucial genetic tests for people with disabilities were those related to lung and colon cancer. However, despite the heightened need and risk in this demographic, the rate of testing for lung and colon cancer through national health checkups was lower compared to other cancer types. This disparity suggests potential missed opportunities for early cancer detection in older adults with disabilities. Therefore, it is imperative for the government to actively devise strategies to enhance national cancer screening rates among people with disabilities. Such measures could pave the way for people with disabilities to lead lives of equitable health and well‐being.

## AUTHOR CONTRIBUTIONS

**Gwanwook Bang:** Conceptualization (equal); methodology (lead); visualization (lead); writing – original draft (lead). **Minji Park:** Methodology (equal); writing – review and editing (supporting). **Jeong‐yeon Seon:** Data curation (lead); formal analysis (lead). **So‐Youn Park:** Conceptualization (equal); project administration (lead); supervision (lead); writing – review and editing (lead).

## FUNDING INFORMATION

This research was supported by the National Research Foundation of Korea (NRF‐2019S1A5A2A03051412). The funders had no role in the study's design, in the collection, analysis, or interpretation of data, in the writing of the manuscript, or in the decision to publish the results.

## CONFLICT OF INTEREST STATEMENT

The authors declare no conflict of interest.

## ETHICAL APPROVAL AND PATIENT CONSENT

This study complied with the Guidelines on De‐Identification of Personal Data of Korea and was approved by the Institutional Review Board of Kyung Hee University [IRB No. KHSIRB‐20‐074(EA)] as a review exemption. Given that the study used de‐identified data, the board waived the requirement for obtaining informed consent from the participants.

## Data Availability

The datasets used and analyzed during this study are available upon reasonable request. The corresponding author should be contacted for the request to access the raw data.

## LIST OF CANCER‐RELATED GENETIC TESTS REGISTERED WITH NATIONAL HEALTH INSURANCE BENEFITS

**Table:**

No.	Test
1	Genetic Tests for Germline Variants_Basic Target Amplification[F8 Gene]
2	Genetic Tests for Germline Variants_PCR‐Extended_PCR‐Hybridization[CYP2C9 Gene]
3	Genetic Tests for Germline Variants_PCR‐Extended_PCR‐Hybridization[F2 Gene]
4	Genetic Tests for Germline Variants_PCR‐Extended_PCR‐Hybridization[F5 Gene]
5	Genetic Tests for Germline Variants_PCR‐Extended_PCR‐Hybridization[MTHFR Gene]
6	Genetic Tests for Germline Variants_PCR‐Extended_PCR‐Hybridization[TPMT Gene]
7	Genetic Tests for Germline Variants_PCR‐Extended_PCR‐Hybridization[VKORC1 Gene]
8	Genetic Tests for Germline Variants_PCR‐Extended_PCR‐Hybridization[TGFBI Gene]
9	Genetic Tests for Germline Variants_PCR‐Extended_PCR‐Fragment Analysis _PCR‐PAGE[ATXN8 Gene]
10	Genetic Tests for Germline Variants_PCR‐Extended_PCR‐Fragment Analysis _PCR‐PAGE[TBP Gene]
11	Genetic Tests for Germline Variants_Sequencing [ELANE Gene]
12	Genetic Tests for Germline Variants_Sequencing [SRD5A2 Gene]
13	Genetic Tests for Germline Variants_Sequencing [TTR Gene]
14	Genetic Tests for Germline Variants_Sequencing [AVP Gene]
15	Genetic Tests for Germline Variants_Sequencing [KCNJ11 Gene]
16	Genetic Tests for Germline Variants_Sequencing [KRAS Gene]
17	Genetic Tests for Germline Variants_Sequencing [NPC2 Gene]
18	Genetic Tests for Germline Variants_Sequencing [PHOX2B Gene]
19	Genetic Tests for Germline Variants_Sequencing [POU3F4 Gene]
20	Genetic Tests for Germline Variants_Sequencing [RNF213 Gene, p.R4810K]
21	Genetic Tests for Germline Variants_Sequencing [RPS19 Gene]
22	Genetic Tests for Germline Variants_Sequencing [SBDS Gene]
23	Genetic Tests for Germline Variants_Sequencing [SDHD Gene]
24	Genetic Tests for Germline Variants_Sequencing [TOR1A gene]
25	Genetic Tests for Germline Variants_Sequencing [ACVRL1 Gene]
26	Genetic Tests for Germline Variants_Sequencing [ALDOB Gene]
27	Genetic Tests for Germline Variants_Sequencing [ARG1 Gene]
28	Genetic Tests for Germline Variants_Sequencing [ARSA Gene]
29	Genetic Tests for Germline Variants_Sequencing [DHCR7 Gene]
30	Genetic Tests for Germline Variants_Sequencing [F7 Gene]
31	Genetic Tests for Germline Variants_Sequencing [G6PC Gene]
32	Genetic Tests for Germline Variants_Sequencing [GALE Gene]
33	Genetic Tests for Germline Variants_Sequencing [GALK1 Gene]
34	Genetic Tests for Germline Variants_Sequencing [GFAP Gene]
35	Genetic Tests for Germline Variants_Sequencing [GLA Gene]
36	Genetic Tests for Germline Variants_Sequencing [HAX1 Gene]
37	Genetic Tests for Germline Variants_Sequencing [IL2RG Gene]
38	Genetic Tests for Germline Variants_Sequencing [MEN1 Gene]
39	Genetic Tests for Germline Variants_Sequencing [NR0B1 Gene]
40	Genetic Tests for Germline Variants_Sequencing [PANK2 Gene]
41	Genetic Tests for Germline Variants_Sequencing [PRF1 Gene]
42	Genetic Tests for Germline Variants_Sequencing [SLC22A12 Gene]
43	Genetic Tests for Germline Variants_Sequencing [SLC2A1 Gene]
44	Genetic Tests for Germline Variants_Sequencing [SLC37A4 Gene]
45	Genetic Tests for Germline Variants_Sequencing [TGFB1 Gene]
46	Genetic Tests for Germline Variants_Sequencing [ACADS Gene]
47	Genetic Tests for Germline Variants_Sequencing [AVPR2 Gene]
48	Genetic Tests for Germline Variants_Sequencing [COL10A1 Gene]
49	Genetic Tests for Germline Variants_Sequencing [CYP17A1 Gene]
50	Genetic Tests for Germline Variants_Sequencing [EDA Gene]
51	Genetic Tests for Germline Variants_Sequencing [EPOR Gene]
52	Genetic Tests for Germline Variants_Sequencing [IKBKG Gene]
53	Genetic Tests for Germline Variants_Sequencing [MAT1A Gene]
54	Genetic Tests for Germline Variants_Sequencing [PRRT2 Gene]
55	Genetic Tests for Germline Variants_Sequencing [RUNX2 Gene]
56	Genetic Tests for Germline Variants_Sequencing [SDHB Gene]
57	Genetic Tests for Germline Variants_Sequencing [SOX9 Gene]
58	Genetic Tests for Germline Variants_Sequencing [SLC7A7 Gene]
59	Genetic Tests for Germline Variants_Sequencing [STAR Gene]
60	Genetic Tests for Germline Variants_Sequencing [TGFBR1 Gene]
61	Genetic Tests for Germline Variants_Sequencing [THRB Gene]
62	Genetic Tests for Germline Variants_Sequencing [ACTA2 Gene]
63	Genetic Tests for Germline Variants_Sequencing [PTS Gene]
64	Genetic Tests for Germline Variants_Sequencing [GATA3 Gene]
65	Genetic Tests for Germline Variants_Sequencing [ABCD1 Gene]
66	Genetic Tests for Germline Variants_Sequencing [ASL Gene]
67	Genetic Tests for Germline Variants_Sequencing [CYBB Gene]
68	Genetic Tests for Germline Variants_Sequencing [ENG Gene]
69	Genetic Tests for Germline Variants_Sequencing [FAH Gene]
70	Genetic Tests for Germline Variants_Sequencing [FUS Gene]
71	Genetic Tests for Germline Variants_Sequencing [GALC Gene]
72	Genetic Tests for Germline Variants_Sequencing [GALT Gene]
73	Genetic Tests for Germline Variants_Sequencing [GBA Gene]
74	Genetic Tests for Germline Variants_Sequencing [GLUD1 Gene]
75	Genetic Tests for Germline Variants_Sequencing [IDUA Gene]
76	Genetic Tests for Germline Variants_Sequencing [IVD Gene]
77	Genetic Tests for Germline Variants_Sequencing [LMNA Gene]
78	Genetic Tests for Germline Variants_Sequencing [MCCC1 Gene]
79	Genetic Tests for Germline Variants_Sequencing [PAH Gene]
80	Genetic Tests for Germline Variants_Sequencing [PAX6 Gene]
81	Genetic Tests for Germline Variants_Sequencing [PCCB Gene]
82	Genetic Tests for Germline Variants_Sequencing [POR Gene]
83	Genetic Tests for Germline Variants_Sequencing [PRODH Gene]
84	Genetic Tests for Germline Variants_Sequencing [PYGM Gene]
85	Genetic Tests for Germline Variants_Sequencing [SALL1 Gene]
86	Genetic Tests for Germline Variants_Sequencing [SLC25A13 Gene]
87	Genetic Tests for Germline Variants_Sequencing [UMOD Gene]
88	Genetic Tests for Germline Variants_Sequencing [ACADM Gene]
89	Genetic Tests for Germline Variants_Sequencing [ALB Gene]
90	Genetic Tests for Germline Variants_Sequencing [F11 Gene]
91	Genetic Tests for Germline Variants_Sequencing [FLCN Gene]
92	Genetic Tests for Germline Variants_Sequencing [FOXP3 Gene]
93	Genetic Tests for Germline Variants_Sequencing [GBE1 Gene]
94	Genetic Tests for Germline Variants_Sequencing [GCDH Gene]
95	Genetic Tests for Germline Variants_Sequencing [GNAS Gene]
96	Genetic Tests for Germline Variants_Sequencing [HADHB Gene]
97	Genetic Tests for Germline Variants_Sequencing [MCCC2 Gene]
98	Genetic Tests for Germline Variants_Sequencing [MPL Gene]
99	Genetic Tests for Germline Variants_Sequencing [MSH6 Gene]
100	Genetic Tests for Germline Variants_Sequencing [MTM1 Gene]
101	Genetic Tests for Germline Variants_Sequencing [MUT Gene]
102	Genetic Tests for Germline Variants_Sequencing [NAGLU Gene]
103	Genetic Tests for Germline Variants_Sequencing [NTRK1 Gene]
104	Genetic Tests for Germline Variants_Sequencing [RAF1 Gene]
105	Genetic Tests for Germline Variants_Sequencing [SGCE Gene]
106	Genetic Tests for Germline Variants_Sequencing [TGFBR2 Gene]
107	Genetic Tests for Germline Variants_Sequencing [SLC3A1 Gene]
108	Genetic Tests for Germline Variants_Sequencing [FANCG Gene]
109	Genetic Tests for Germline Variants_Sequencing [ZEB2 Gene]
110	Genetic Tests for Germline Variants_Sequencing [CBS Gene]
111	Genetic Tests for Germline Variants_Sequencing [MUTYH Gene]
112	Genetic Tests for Germline Variants_Sequencing [G6PD Gene]
113	Genetic Tests for Germline Variants_Sequencing [F12 Gene]
114	Genetic Tests for Germline Variants_Sequencing [HEXA Gene]
115	Genetic Tests for Germline Variants_Sequencing [SLC2A2 Gene]
116	Genetic Tests for Germline Variants_Sequencing [GALNS Gene]
117	Genetic Tests for Germline Variants_Sequencing [TMPRSS6 Gene]
118	Genetic Tests for Germline Variants_Sequencing [HEXB Gene]
119	Genetic Tests for Germline Variants_Sequencing [FRMD7 Gene]
120	Genetic Tests for Germline Variants_Sequencing [AGL Gene]
121	Genetic Tests for Germline Variants_Sequencing [APC Gene]
122	Genetic Tests for Germline Variants_Sequencing [ATP7A Gene]
123	Genetic Tests for Germline Variants_Sequencing [CFH Gene]
124	Genetic Tests for Germline Variants_Sequencing [CPS1 Gene]
125	Genetic Tests for Germline Variants_Sequencing [ELN Gene]
126	Genetic Tests for Germline Variants_Sequencing [GAA Gene]
127	Genetic Tests for Germline Variants_Sequencing [MYH9 Gene]
128	Genetic Tests for Germline Variants_Sequencing [NSD1 Gene]
129	Genetic Tests for Germline Variants_Sequencing [OCRL Gene]
130	Genetic Tests for Germline Variants_Sequencing [PCCA Gene]
131	Genetic Tests for Germline Variants_Sequencing [PEX1 Gene]
132	Genetic Tests for Germline Variants_Sequencing [PHEX Gene]
133	Genetic Tests for Germline Variants_Sequencing [SCN4A Gene]
134	Genetic Tests for Germline Variants_Sequencing [SOS1 Gene]
135	Genetic Tests for Germline Variants_Sequencing [STAT3 Gene]
136	Genetic Tests for Germline Variants_Sequencing [UNC13D Gene]
137	Genetic Tests for Germline Variants_Sequencing [ABCC8 Gene]
138	Genetic Tests for Germline Variants_Sequencing [CAPN3 Gene]
139	Genetic Tests for Germline Variants_Sequencing [F8 Gene]
140	Genetic Tests for Germline Variants_Sequencing [GNPTAB Gene]
141	Genetic Tests for Germline Variants_Sequencing [INSR Gene]
142	Genetic Tests for Germline Variants_Sequencing [MYH7 Gene]
143	Genetic Tests for Germline Variants_Sequencing [NPC1 Gene]
144	Genetic Tests for Germline Variants_Sequencing [PHKA2 Gene]
145	Genetic Tests for Germline Variants_Sequencing [PTCH1 Gene]
146	Genetic Tests for Germline Variants_Sequencing [RP1L1 Gene]
147	Genetic Tests for Germline Variants_Sequencing [SCN1A Gene]
148	Genetic Tests for Germline Variants_Sequencing [SLC12A3 Gene]
149	Genetic Tests for Germline Variants_Sequencing [TCOF1 Gene]
150	Genetic Tests for Germline Variants_Sequencing [VPS33B Gene]
151	Genetic Tests for Germline Variants_Sequencing [CHD7 Gene]
152	Genetic Tests for Germline Variants_Sequencing [COL2A1 Gene]
153	Genetic Tests for Germline Variants_Sequencing [COL3A1 Gene]
154	Genetic Tests for Germline Variants_Sequencing [CREBBP Gene]
155	Genetic Tests for Germline Variants_Sequencing [FANCA Gene]
156	Genetic Tests for Germline Variants_Sequencing [NIPBL Gene]
157	Genetic Tests for Germline Variants_Sequencing [KMT2D Gene]
158	Genetic Tests for Germline Variants_Sequencing [PKD1 Gene]
159	Genetic Tests for Germline Variants_Sequencing [SPG11 Gene]
160	Genetic Tests for Germline Variants_Sequencing [PKHD1 Gene]
161	Genetic Tests for Germline Variants_Southern Blot[D4Z4 Repeat]
162	Genetic Tests for Somatic Variants_PCR‐Extended_Nested PCR[MLL Gene, Partial Tandem Duplication]
163	Genetic Tests for Somatic Variants_PCR‐Extended_PCR‐Hybridization[BAALC Gene]
164	Genetic Tests for Somatic Variants_PCR‐Extended_PCR‐Hybridization[BRAF Gene]
165	Genetic Tests for Somatic Variants_PCR‐Extended_PCR‐Hybridization[CBFB‐MYH11 Fusion Gene]
166	Genetic Tests for Somatic Variants_PCR‐Extended_PCR‐Hybridization[EGFR Gene]
167	Genetic Tests for Somatic Variants_PCR‐Extended_PCR‐Hybridization[KRAS Gene]
168	Genetic Tests for Somatic Variants_PCR‐Extended_PCR‐Hybridization[NPM1 Gene]
169	Genetic Tests for Somatic Variants_PCR‐Extended_PCR‐Hybridization[WT1 Gene]
170	Genetic Tests for Somatic Variants_PCR‐Extended_PCR‐Hybridization[IDH1 Gene]
171	Genetic Tests for Somatic Variants_PCR‐Extended_PCR‐Hybridization[MPL Gene]
172	Genetic Tests for Somatic Variants_PCR‐Extended_PCR‐Hybridization[NRAS Gene]
173	Genetic Tests for Somatic Variants_PCR‐Extended_PCR‐PAGE[TRG Gene]
174	Genetic Tests for Somatic Variants_PCR‐Extended_PCR‐PAGE[TRD Gene]
175	Genetic Tests for Somatic Variants_PCR‐Extended_PCR‐PAGE[TRB Gene]
176	Genetic Tests for Somatic Variants_PCR‐Extended_PCR‐PAGE[IGH Gene]
177	Genetic Tests for Somatic Variants_PCR‐Extended_PCR‐PAGE[IGK Gene]
178	Genetic Tests for Somatic Variants_PCR‐Extended_PCR‐PAGE[IGL Gene]
179	Genetic Tests for Somatic Variants_PCR‐Extended_PCR‐PAGE[FLT3 Gene, Internal Tandem Duplication]
180	Genetic Tests for Somatic Variants_Sequencing [JAK2 Gene, Exon 12]
181	Genetic Tests for Somatic Variants_Sequencing [NPM1 Gene]
182	Genetic Tests for Somatic Variants_Sequencing [BRAF Gene]
183	Genetic Tests for Somatic Variants_Sequencing [IDH1 Gene]
184	Genetic Tests for Somatic Variants_Sequencing [MPL Gene]
185	Genetic Tests for Somatic Variants_Sequencing [IDH2 Gene]
186	Genetic Tests for Somatic Variants_Sequencing [PDGFRA Gene]
187	Genetic Tests for Somatic Variants_Sequencing [CSF3R Gene]
188	Genetic Tests for Somatic Variants_Sequencing [CEBPA Gene]
189	Genetic Tests for Somatic Variants_Sequencing [CALR Gene]
190	Genetic Tests for Somatic Variants_Sequencing [TP53 Gene]
191	Genetic Tests for Somatic Variants_Sequencing [CBL Gene]
192	Genetic Tests for Somatic Variants_Sequencing [RUNX1 Gene]
